# Population pharmacokinetic modeling of CSF to blood clearance: prospective tracer study of 161 patients under work-up for CSF disorders

**DOI:** 10.1186/s12987-022-00352-w

**Published:** 2022-07-01

**Authors:** Markus Herberg Hovd, Espen Mariussen, Hilde Uggerud, Aslan Lashkarivand, Hege Christensen, Geir Ringstad, Per Kristian Eide

**Affiliations:** 1 Section for Pharmacology and Pharmaceutical Biosciences, Department of Pharmacy, University of Oslo, Oslo, Norway; 2 Norwegian Institute for Air Research, Kjeller, Norway; 3 Department of Neurosurgery, Oslo University Hospital—Rikshospitalet, Pb 4950 Nydalen, 0424 Oslo, Norway; 4 Institute of Clinical Medicine, Faculty of Medicine, University of Oslo, Oslo, Norway; 5 Division of Radiology and Nuclear Medicine, Department of Radiology, Oslo University Hospital—Rikshospitalet, Oslo, Norway; 6 Department of Geriatrics and Internal Medicine, Sorlandet Hospital, Arendal, Norway; 7 Department of Air Quality and Noise, Norwegian Institute of Public Health, Oslo, Norway

**Keywords:** Cerebrospinal fluid, Clearance, Gadobutrol, Brain metabolites, Intrathecal administration, Intrathecal drugs

## Abstract

**Background:**

Quantitative measurements of cerebrospinal fluid to blood clearance has previously not been established for neurological diseases. Possibly, variability in cerebrospinal fluid clearance may affect the underlying disease process and may possibly be a source of under- or over-dosage of intrathecally administered drugs. The aim of this study was to characterize the cerebrospinal fluid to blood clearance of the intrathecally administered magnetic resonance imaging contrast agent gadobutrol (Gadovist, Bayer Pharma AG, GE). For this, we established a population pharmacokinetic model, hypothesizing that cerebrospinal fluid to blood clearance differs between cerebrospinal fluid diseases.

**Methods:**

Gadobutrol served as a surrogate tracer for extra-vascular pathways taken by several brain metabolites and drugs in cerebrospinal fluid. We estimated cerebrospinal fluid to blood clearance in patients with different cerebrospinal fluid disorders, i.e. symptomatic pineal and arachnoid cysts, as well as tentative spontaneous intracranial hypotension due to cerebrospinal fluid leakage, idiopathic intracranial hypertension, or different types of hydrocephalus (idiopathic normal pressure hydrocephalus, communicating- and non-communicating hydrocephalus). Individuals with no verified cerebrospinal fluid disturbance at clinical work-up were denoted references.

**Results:**

Population pharmacokinetic modelling based on 1,140 blood samples from 161 individuals revealed marked inter-individual variability in pharmacokinetic profiles, including differences in absorption half-life (time to 50% of tracer absorbed from cerebrospinal fluid to blood), time to maximum concentration in blood and the maximum concentration in blood as well as the area under the plasma concentration time curve from zero to infinity. In addition, the different disease categories of cerebrospinal fluid diseases demonstrated different profiles.

**Conclusions:**

The present observations of considerable variation in cerebrospinal fluid to blood clearance between individuals in general and across neurological diseases, may suggest that defining cerebrospinal fluid to blood clearance can become a useful diagnostic adjunct for work-up of cerebrospinal fluid disorders. We also suggest that it may become useful for assessing clearance capacity of endogenous brain metabolites from cerebrospinal fluid, as well as measuring individual cerebrospinal fluid to blood clearance of intrathecal drugs.

## Background

While the renal glomerular filtration rate (GFR) is clinically used as marker of clearance of drugs and solutes from blood [1], the cerebrospinal fluid (CSF) to blood clearance has not previously been defined in either healthy individuals nor in individuals with neurological diseases. Possibly, direct measurement of CSF to blood clearance might be useful for understanding diseases of the brain, and consequently lay ground for personalized intrathecal drug administration to the central nervous system (CNS).

Since the dual discoveries of the glymphatic system in 2012 [2] and the meningeal lymphatic system in 2015 [3], there have been renewed interest in how various waste substances are cleared from the brain [4], and not at least the role of meningeal lymphatic vessels [5]. Impaired glymphatic clearance of toxic by-products from brain metabolism to CSF causing deposition of toxic substances in the brain, e.g. deposition of amyloid-β and tau in Alzheimer’s disease and α-synuclein in Parkinson’s disease, has been proposed as a common pathogenic pathway behind several neurodegenerative disorders [4]. Meningeal lymphatic function seems to be affected in a wide range of diseases, as indicated in animal models of traumatic brain injury [6], malignant brain disease [7–9], stroke [10, 11] and Alzheimer’s disease [12], and in patients with Parkinson’s disease[13]. Given that impaired molecular clearance from CSF to blood may have a pivotal role in the development of neurological disease; it might be desirable to obtain quantitative data about CSF to blood clearance on an individual basis. For years, levels of brain metabolites from single time points have been measured in CSF, as well as in blood, aiming at identifying the pre-symptomatic phase of dementia disease [14, 15]. On the other hand, direct assessment of clearance dynamics from CSF to blood has not been possible.

Assessment of CSF to blood clearance might as well be useful to tailor dosage of intrathecal drugs. Today, intrathecal drug administration seems promising in order to treat a wide range of diseases within the CNS, such as neuro-inflammatory, neuro-degenerative, neuro-oncologic, and neuro-vascular diseases [16–20]. Many systemically administered drugs, which are supposed to function in CNS, remain to a considerable degree within the systemic circulation due to their inability to cross the blood–brain-barrier (BBB) [21]. Given previous observations of brain wide distribution of CSF tracer administered to the lumbar subarachnoid space in humans [22], intrathecally administered drugs have potential to better target brain disease directly by their by-passing of the BBB, and assumedly in much lower doses than applied systemically, thereby reducing side effects.

Our group has used intrathecal administration of the magnetic resonance imaging (MRI) contrast agent gadobutrol (serving as a CSF tracer) to explore molecular passage from CSF to the brain [22, 23], meninges [24], calvarial bone [25], extra-cranial lymph nodes [26], as well as to the blood [27]. From this, we suggest that measurements of CSF to blood clearance of gadobutrol may provide an overall estimation of the ability of CSF to remove macromolecules. Since tracer levels in blood are highly correlated with levels of tracer in CSF at MRI [27], resource-demanding imaging may be omitted as part of CSF clearance assessment. Gadobutrol is a hydrophilic substance unable to cross the BBB, which after administration to CSF is excreted along the same pathways as other endogenous substances within CSF, such as the paravascular [4] and meningeal lymphatic pathways [28] suggested from animal studies. In the present work, we investigated the CSF to blood clearance of gadobutrol in patients under clinical work-up of various neurological diseases and CSF disturbances, employing a population pharmacokinetic model based on a large patient material spanning multiple disease categories. The hypothesis was that different CSF diseases present a characteristic profile of CSF to blood clearance.

## Methods

### Experimental design

A prospective and observational study design was utilized; randomization or a priori sample size calculation was not relevant.

### Patients

The study included patients referred to the Department of neurosurgery, Oslo University Hospital—Rikshospitalet, Oslo, Norway, who were examined for tentative CSF disorders, and in whom intrathecal contrast enhanced MRI was considered indicated for clinical reasons. Individuals who were not eligible for inclusion included subjects with a history of hypersensitivity reactions to contrast media agents, severe allergic reactions in general, evidence of renal dysfunction, i.e. glomerular filtration rate (GFR) < 30, age < 18 or > 80 years, or pregnant or breastfeeding women.

Patients were categorized according to tentative diagnosis prior to MRI, and underwent work-up, including blood sampling, prior to any treatment. The category *reference subjects* (REF) includes individuals in whom we found no apparent evidence of CSF disturbance and no indication for surgery. The group with spontaneous intracranial hypotension (SIH) had an identified CSF leakage that required surgery to close the leakage. The present subjects in the category idiopathic intracranial hypertension (IIH) were shunted and demonstrated clinical improvement thereafter. Patients with pineal cysts (PC) or arachnoid cysts (AC) underwent surgery with cyst removal and demonstrated post-operative clinical improvement. The category idiopathic normal pressure hydrocephalus (iNPH) included patients who based on clinical workup, imaging findings and results of intracranial pressure (ICP) monitoring [29, 30], underwent shunting with a demonstrated clinical improvement thereby qualifying for the diagnosis Definite iNPH according to the Japanese guidelines [31].

### Intrathecal administration of gadobutrol

The MRI contrast agent gadobutrol (Gadovist™, Bayer Pharma AG, Berlin, Germany) was administered intrathecally in volumes of 0.10, 0.25 or 0.5 mL (1.0 mmol/mL) at a speed of a few seconds. The intrathecal injection procedure was done at the lumbar level. Correct entrance to the subarachnoid space was verified by CSF backflow from the spinal needle.

The first 80 patients received intrathecal gadobutrol in a dose of 0.50 mmol only, and the latter patients received intrathecal gadobutrol in alternatig doses of 0.10 mmol, 0.25 mmol or 0.5 mmol.

### Quantification of gadolinium in blood

Venous blood samples were obtained at empirically determined regular time points up to about 48 h after intrathecal administration of gadobutrol, and were stored at -80 °C. Quantification of gadolinium to estimate concentrations of gadobutrol in blood and plasma was performed as previously described [27]. In short, the whole blood samples were homogenized using an Ultra-Turrax homogenizer (IKA T18). Both plasma and the homogenized whole blood samples were subjected to digestion with ultrapure distilled nitric acid and deionized Milli-Q water in a closed-vessel microwave technique system (UltraCLAVE, Milestone, Italy). The samples were digested according to a 60-min stepwise heating program, with a maximum temperature of 250 °C held for 15 min. Following dilution, samples were analyzed for gadolinium by inductively coupled plasma mass spectrometry (Agilent 7700x, Agilent Technologies), employing indium at 0.1 μg/L as an internal standard. A 5-point standard curve (0.01–10 μg/L) was used. All analytical results were corrected for procedural blank values. Measured gadolinium concentrations were recalculated to gadobutrol concentrations.

In this work, both plasma and whole-blood gadobutrol were used. Linear regression through the origin was used to determine the plasma to whole blood ratio, and whole blood concentration of gadobutrol was interpolated to plasma concentrations for the purpose of pharmacokinetic modelling.

### Gadobutrol population pharmacokinetic modelling

A population pharmacokinetic model was developed in order to determine individual pharmacokinetic parameters of intrathecally administered gadobutrol. A non-parametric adaptive grid approach implemented in Pmetrics (version 1.9.7) for R (version 4.0.0) was used [32]. Based on available literature [33, 34] and previous work [27], both one- and two-compartment structural models were initially considered. The structural models provide the hypothesized framework for which transfer of gadobutrol occurs between compartments. Both the one- and two-compartmental models estimate the transfer of gadobutrol from CSF to blood, and elimination from blood. However, in the two-compartmental model, a peripheral tissue compartment was implemented, allowing gadobutrol to distribute into and from tissue. For the purpose of internal model validation, the dataset was split into a development- (80%) and validation-set (20%). Patients with more than six samples were allocated to the development set, with additional random allocation of profiles until 80% of total profiles. Model selection was primarily based on comparison of the relative root mean squared predictive error (RMSE, %) calculated from the relative predictive error of all gadobutrol concentrations in the development dataset. Additionally, the linear regression slope, R^2^-values of the observed versus predicted plots, Akaike’s information criteria (AIC) and the Bayesian information criteria (BIC) also guided model development to some extent. Covariates were not included, due to sole interest in individual predictions.

### Pharmacokinetic calculations

Posterior individual parameter values, as well as posterior individually predicted concentrations obtained from the final population pharmacokinetic model run with the complete dataset, were used for all pharmacokinetic calculations. Predictions were made in one-minute intervals from time of administration and up to 72 h. The following pharmacokinetic variables were evaluated:

The absorption half-life (T_1/2, abs_) is defined as the time for half the amount of gadobutrol in the CSF to be cleared to blood. This parameter was used as a surrogate marker for CSF to blood clearance of gadobutrol. T_1/2, abs_ was calculated by dividing the natural logarithm of 2 over the model-estimated coefficient of absorption (*K*_*a*_) from CSF to blood.

Time to maximum concentration (T_max_) in plasma and maximum concentration (C_max_) in plasma were obtained directly from the individual predictions.

Lag-time of absorption to blood (T_lag_) is the model-estimated time for the tracer to reach the site of clearance in CSF. Longer T_lag_ thus implies that the molecule stays longer within CSF or that it takes longer before the clearance process to blood starts.

Area under the concentration–time curve from zero to infinity (AUC_0-∞_) was calculated with the trapezoidal approximation from individual posterior predicted concentrations using the ‘makeAUC’-function in the Pmetrics package for R. The AUC is a measure of systemic exposure of gadobutrol.

In order to compare parameters across multiple doses, C_max_ and AUC_0-∞_ were normalized with respect to dose.

### Statistical analysis

Comparisons between groups were performed using two-tailed individual samples t-test for continuous variables, and Fishers exact test for categorical variables. Values were visually assessed for normality prior to testing. Differences in parameters and normalized parameters between different doses were assessed using an analysis of variance. For the predefined α = 0.05, we considered 95% confidence intervals not including zero and P-values lower than 0.05 to be statistically significant.

## Results

### Patient material

The study included 161 patients, with a mean ± SD age of 54 ± 19 years (range 19 to 82 years), and with a mean body mass index (BMI) of 28 ± 5 kg/m^2^ (range 18 to 41 kg/m^2^). Patients were under clinical work-up for possible CSF disorders, with diagnosis categories as indicated in Table 1.

**Table 1:** Demographic overview

	Patient category							
	REF	PC	AC	SIH	IIH	iNPH	Comm. HC	Non-comm. HC
Number of subjects	28	13	14	14	15	63	11	3
Ith dose of gadobutrol								
0.10 mmol	0	0	0	0	0	13	0	0
0.25 mmol	3	0	2	4	1	17	3	0
0.50 mmol	25	13	12	10	14	33	8	3
Gender (male/female)	6/22	1/12	8/6^a^	5/9	2/13	37/26^b^	7/4^a^	2/1
Age (years)	39 ± 12	36 ± 13	52 ± 17^a^	50 ± 10^b^	33 ± 11	72 ± 6^c^	49 ± 13^a^	43 ± 29
Height (cm)	172 ± 8	170 ± 5	176 ± 10	172 ± 10	165 ± 7^b^	173 ± 9	178 ± 12	171 ± 7
Weight (kg)	82 ± 15	80 ± 15	82 ± 13	78 ± 23	88 ± 17	81 ± 16	84 ± 20	80 ± 24
BMI (kg/m^2^)	28 ± 5	28 ± 4	27 ± 3	26 ± 6	32 ± 5^a^	27 ± 4	26 ± 5	27 ± 6
GFR (ml/min)	103 ± 12	98 ± 12	86 ± 16^b^	95 ± 15	105 ± 13	77 ± 14^c^	92 ± 18	104 ± 15

Data presented as mean ± SD. Differences from the reference group were determined by independent samples t-test for continuous variables and by Fishers exact test for categorical variables (^a^P < 0.05, ^b^P < 0.01, ^c^P < 0.001). Patient categories: *AC* Arachnoid cyst, *Comm HC* communicating hydrocephalus, *IIH* idiopathic intracranial hypertension, *iNPH* idiopathic normal pressure hydrocephalus, *Non-comm HC* non-communicating hydrocephalus, *PC* pineal cyst, *REF* reference cohort, *SIH* spontaneous intracranial hypotension

Several groups were statistically significantly different from the reference (REF) cohort, with respect to gender, age, height, body mass index (BMI), and kidney function. A total of 1,140 samples were analyzed for gadobutrol in plasma or whole blood; the mean number of samples was 8 ± 2 in each subject (range 1 to 11 samples).

### Gadobutrol blood-to-plasma ratio

**Fig. 1:**
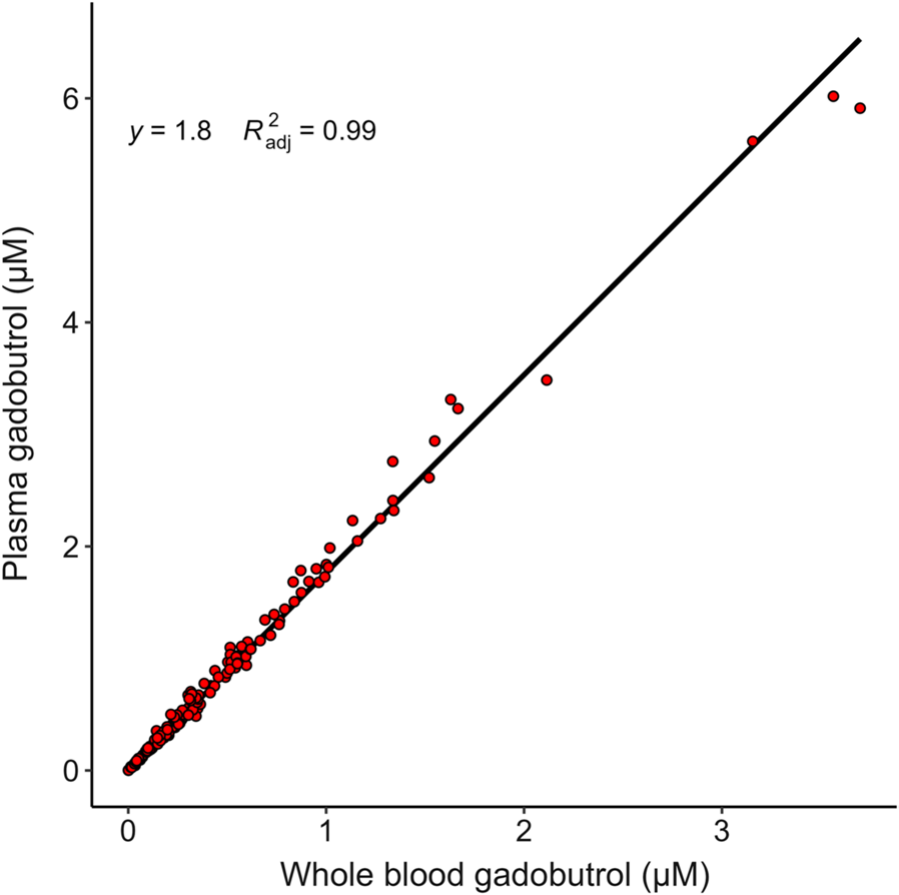
Whole blood to plasma gadobutrol. Figure demonstrates linear regression through the origin of whole-blood to plasma gadobutrol (μM). The black line represents the linear curve with formula *y* = *1.795x*

In 24 patients, 204 samples were concomitantly analyzed for gadobutrol in both plasma and whole blood. Concentration of gadobutrol in plasma was linearly associated (β = 1.795, *R*^*2*^_*adjusted*_ = 0.997; P < 0.001) with whole blood concentration of gadobutrol (Fig. 1), and whole blood concentration of gadobutrol was interpolated to plasma concentrations for the purpose of pharmacokinetic modelling, using linear regression through the origin.

### Population pharmacokinetic modeling

Both one- and two-compartment models were initially evaluated. Compared to the one-compartment structural model, a two-compartment model improved the goodness of fit. Furthermore, addition of an absorption lag-time improved the individual predictions, especially in the absorption phase.

**Fig. 2:**
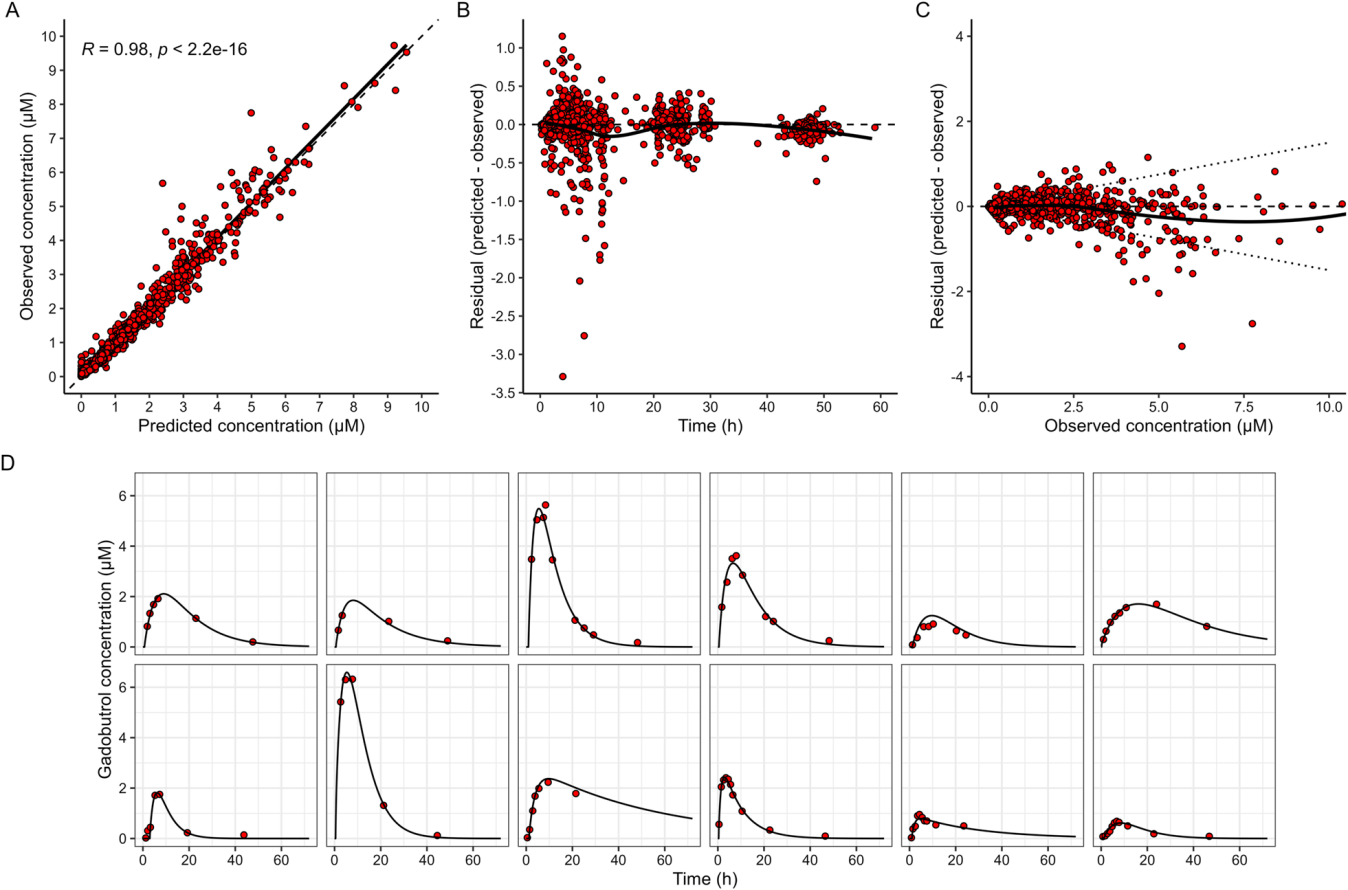
Population pharmacokinetic model diagnostic plots. **A** Observed gadobutrol concentrations against posterior individual predicted concentrations. Dashed and solid lines represent the unity and linear regression line, respectively. **B** Residual error against time, solid line represents the l. **C** Residual error against individual observed gadobutrol concentrations, dotted lines represent the treshold for 15% relative error. **D** Randomly selected patient profiles, with observations (dot) and individual predictions (solid line), demonstrating goodness of fit

The final population pharmacokinetic model consisted of two compartments with first-order transfer from CSF to blood, and first-order elimination from the central compartment (blood) and absorption lag-time, and the model described the data well (Fig. 2A). The final model ran on the complete dataset achieved a mean prediction error of -0.032, a root mean squared error of 0.283, and a percentage root mean squared error of 18.5%. Akaike’s Information Criteria (AIC) and Bayesian Information Criteria (BIC) were 667 and 702, respectively. When assessing residual error for different times, a trend for underprediction was shown during times between 5 and 15 h (Fig. 2B). No systematic trends were found when comparing residual error to the observed concentration of gadobutrol (Fig. 2C). Individual predictions for a random subset of patients are shown in Fig. 2D, demonstrating goodness of fit.

### Dose linearity

**Fig. 3:**
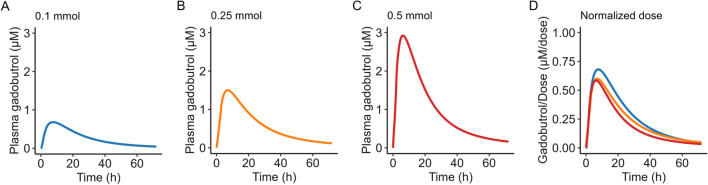
Dose linearity of intrathecally administered gadobutrol. Mean individual posterior predicted concentration of gadobutrol following intrathecal administration of **A** 0.1 mmol, **B** 0.25 mmol and **C** 0.5 mmol of gadobutrol; also shown is the **D** dose-normalized concentrations, demonstrating dose linearity

Mean pharmacokinetic profiles across intrathecally-administered doses of gadobutrol are shown in Fig. 3. No differences in neither absorption half-life, time to maximum concentration, nor dose-normalized maximum concentration were found across the administered doses of gadobutrol. However, a statistically significant difference in dose-normalized AUC_0-∞_ between the dose levels of 0.1 mmol and 0.5 mmol was found (Δ = − 5.22 [95% CI: − 9.68, − 0.77] μM h). Additionally, mean predictive error of the population pharmacokinetic model was not different between dose levels.

### Inter-individual variability in gadobutrol CSF to blood clearance

**Fig. 4:**
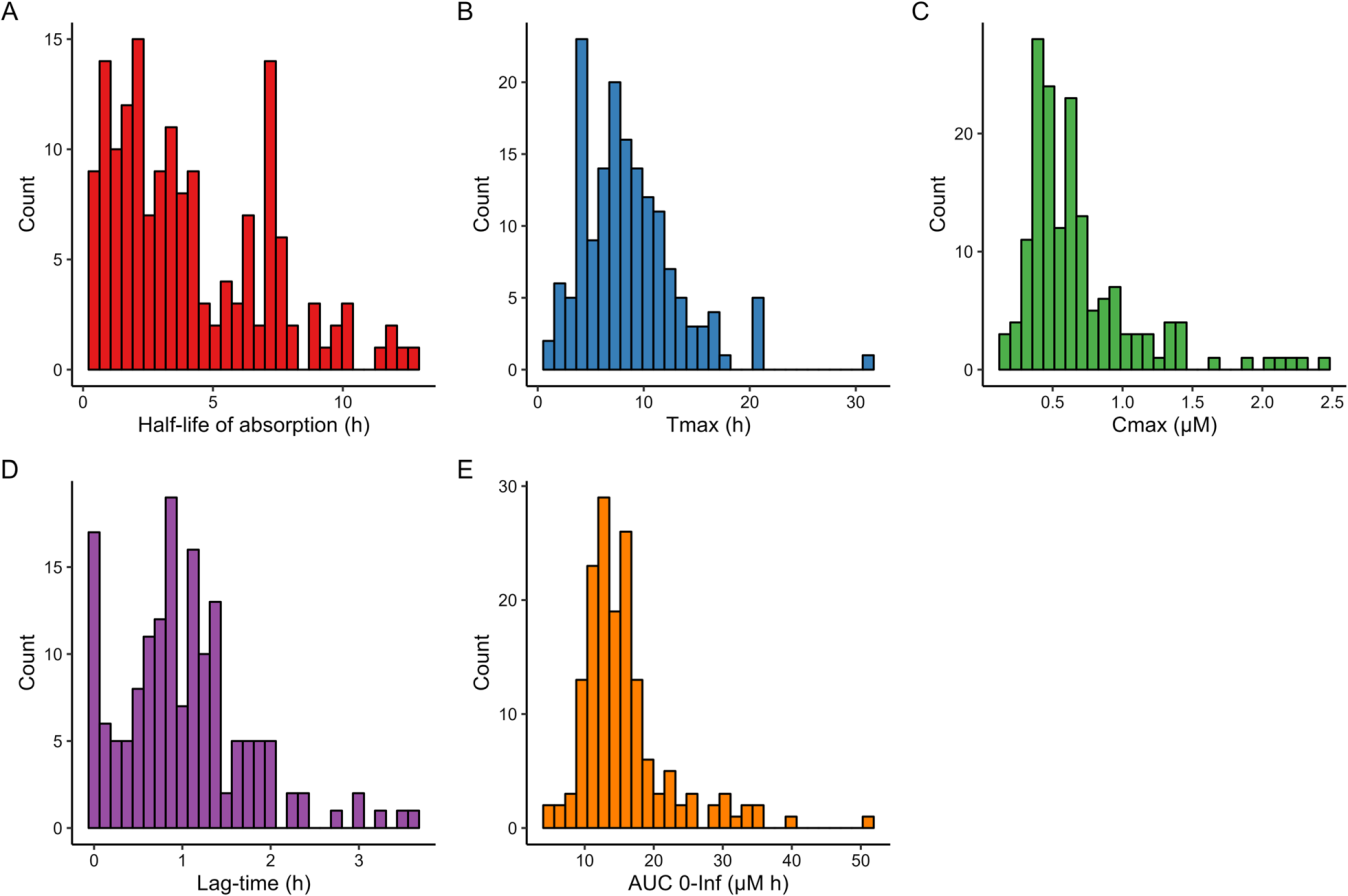
Distribution of indivdual pharmacokinetic parameters for entire cohort. Histogram of parameter distribution for the **A** absorption half-life (T_1/2, abs_), **B** time to maximum concentration (T_max_), **C** maximum concentration (C_max_), **D** lag-time (T_lag_), and **E** area under the curve (AUC) from zero to infinity for the entire cohort of patients (n = 161)

Irrespective of diagnosis category, a large degree of inter-individual variability was observed with respect to the pharmacokinetic parameters of intrathecally administered gadobutrol. For the complete dataset, mean absorption half-life was 3.83 ± 2.50 h, with a coefficient of variation (CV) of 65%, which did not vary with dose. Time to maximum concentration (T_max_) and dose-normalized maximum concentration (C_max_) were 8.60 ± 4.58 h (CV 53%) and 0.69 ± 0.42 μM (CV 61%) respectively. The large inter-individual variability of pharmacokinetic parameters irrespective of diagnosis is shown in Fig. 4.

### Disease categories show different profiles

**Fig. 5:**
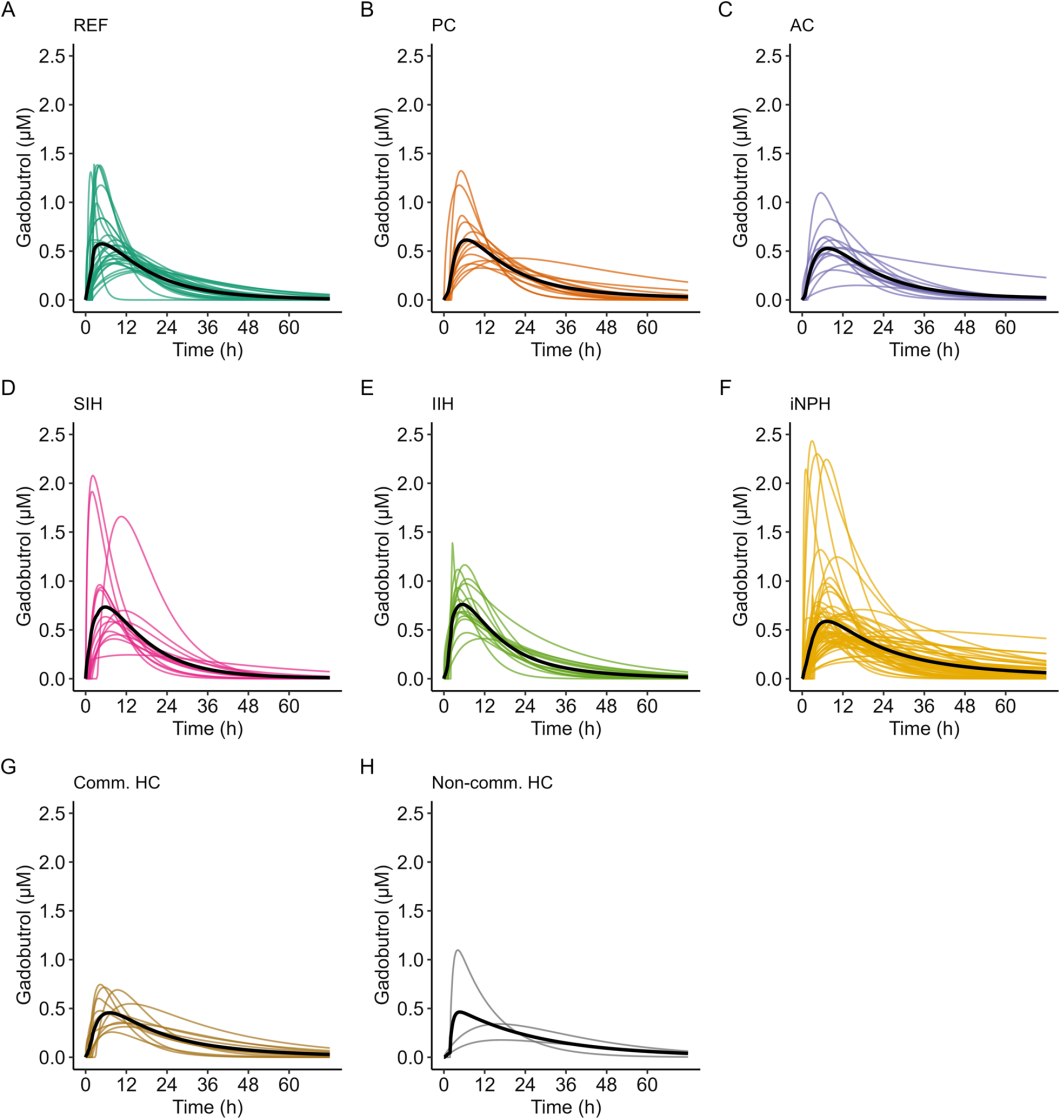
Inter-individual variation in CSF to blood clearance within each diagnosis category. Individual posterior dose-normalized predicted concentrations of gadobutrol over time for the **A** reference (REF), **B** pineal cyst (PC), **C** arachnoid cyst (AC), **D** spontaneous intracranial hypotension (SIH), **E** idiopathic intracranial hypertension (IIH), **F** idiopathic normal pressure hydrocephalus (iNPH), **G** communicating hydrocephalus (Comm HC) and **H** non-communicating hydrocephalus (Non-comm HC) groups. Black lines represent the mean concentration for each group, averaged at each time-point

**Fig. 6:**
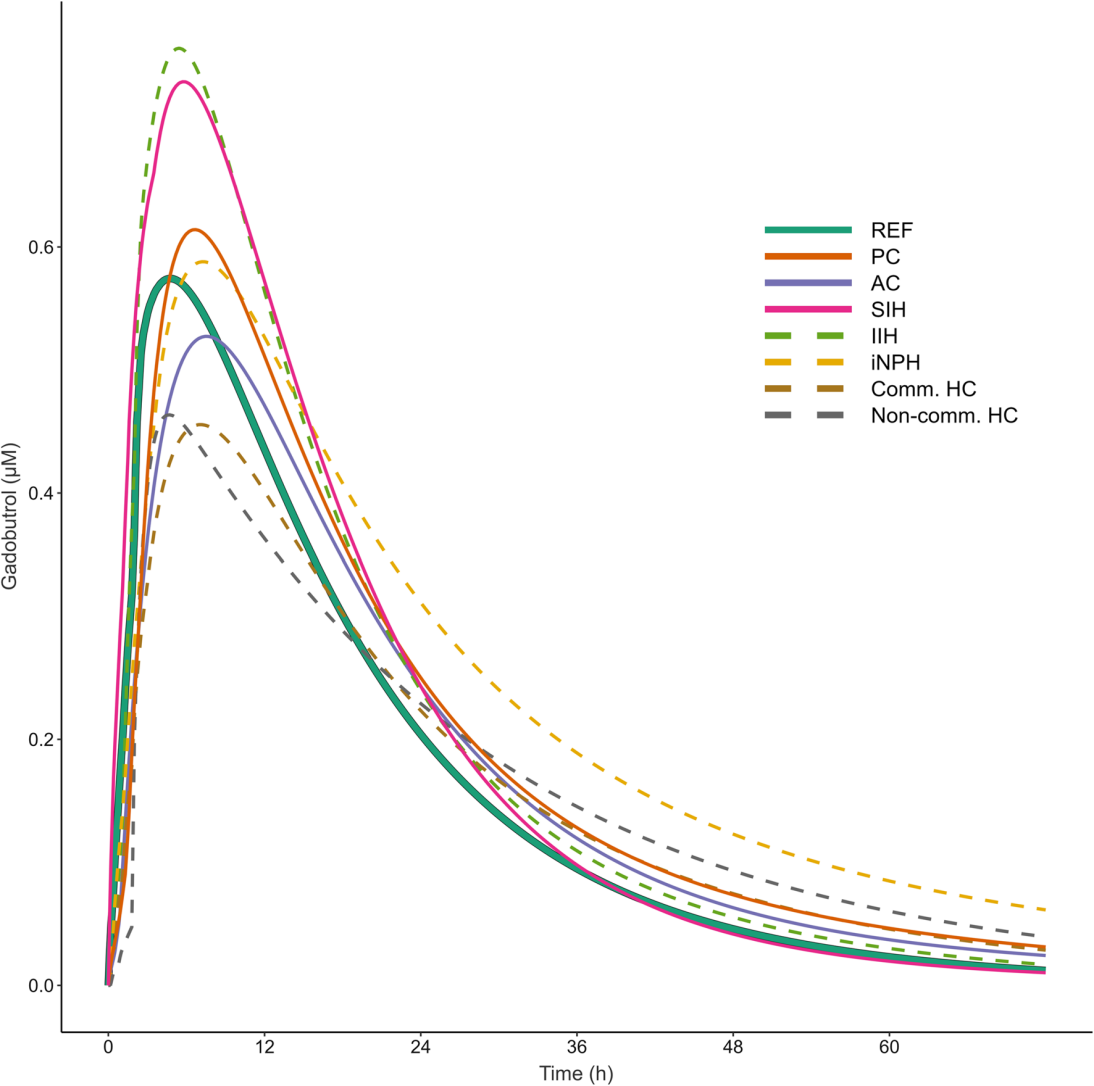
Mean concentration profiles of gadobutrol for the different patient groups. Individual posterior predicted dose-normalized blood concentrations of intrathecal gadobutrol from the population pharmacokinetic model, averaged at each time-point by group. The reference group is highlighted by a thick solid line. *REF* Reference, *PC* pineal cyst, *AC* arachnoid cyst, *SIH* Spontaneous intracranial hypotension (SIH), *IIH* idiopathic intracranial hypertension, *iNPH* idiopathic normal pressure hydrocephalus, *Comm HC* communicating hydrocephalus, *Non-comm HC* non-communicating hydrocephalus

A notable degree of variability in pharmacokinetic parameters was observed both within and between disease categories. Individual predicted profiles with group-wise mean predicted profiles are shown in Fig. 5, and pharmacokinetic parameters at group level with comparisons are presented in Table 2. Variability in mean concentration profiles of gadobutrol for the different patient groups is further presented in Fig. 6, illustrating the between group differences.

**Table 2:** Model predicted pharmacokinetic parameters of gadobutrol in blood

	Patient category							
	REF	PC	AC	SIH	IIH	iNPH	Comm. HC	Non-comm. HC
Number of subjects	28	13	14	14	15	63	11	3
T_1/2, abs_ (h)	4.57 ± 3.31 (72%)	4.12 ± 2.14 (52%)	4.86 ± 2.93 (60%)	3.79 ± 2.91 (77%)	2.32 ± 1.61^b^ (69%)	4.15 ± 3.07 (74%)	4.62 ± 3.86 (84%)	4.92 ± 3.88 (79%)
T_max_ (h)	7.49 ± 4.09 (55%)	9.00 ± 4.27 (47%)	8.89 ± 2.98 (34%)	7.09 ± 3.44 (49%)	5.8 ± 2.01 (35%)	9.85 ± 5.4 ^a^ (55%)	8.14 ± 3.44 (42%)	12.33 ± 7.17 (58%)
C_max_ (μM)	0.70 ± 0.38 (54%)	0.66 ± 0.31 (47%)	0.55 ± 0.23 (42%)	0.90 ± 0.58 (64%)	0.83 ± 0.27 (33%)	0.67 ± 0.48 (72%)	0.50 ± 0.17 ^a^ (34%)	0.54 ± 0.49 (91%)
T_lag_ (h)	0.74 ± 0.67 (91%)	1.20 ± 0.59 ^a^ (49%)	0.74 ± 0.42 (57%)	1.03 ± 0.89 (86%)	0.88 ± 0.55 (62%)	1.16 ± 0.77 ^a^ (66%)	1.12 ± 0.87 (78%)	0.96 ± 0.82 (85%)
AUC_0-∞_ (μM h)	12.58 ± 2.55 (20%)	15.05 ± 3.96 (26%)	13.65 ± 6.13 (45%)	15.36 ± 5.92 (39%)	15.67 ± 4.54^a^ (29%)	18.49 ± 8.24^c^ (45%)	12.79 ± 4.68 (37%)	13.44 ± 3.6 (27%)

Data presented as mean ± SD (coefficient of variation given in parenthesis). Abbreviations: T_1/2, abs_ = Time to 50% of tracer dose absorbed to blood (absorption half-life), indicative of CSF tracer clearance to blood. T_max_ = Time to maximum concentration. C_max_ = Dose-normalized maximum concentration. T_lag_ = lag-time of absorption. AUC_0-∞_ = Dose-normalized area under curve from zero to infinity. Significant difference from REF: ^a^P < 0.05, ^b^P < 0.01, ^c^P < 0.001 (independent samples t-test). Patient categories: *AC* arachnoid cyst, *Comm HC* communicating hydrocephalus, *IIH* idiopathic intracranial hypertension, *iNPH*: idiopathic normal pressure hydrocephalus, *Non-comm HC* non-communicating hydrocephalus, *PC* pineal cyst, *REF* reference cohort, *SIH* spontaneous intracranial hypotension

**Fig. 7:**
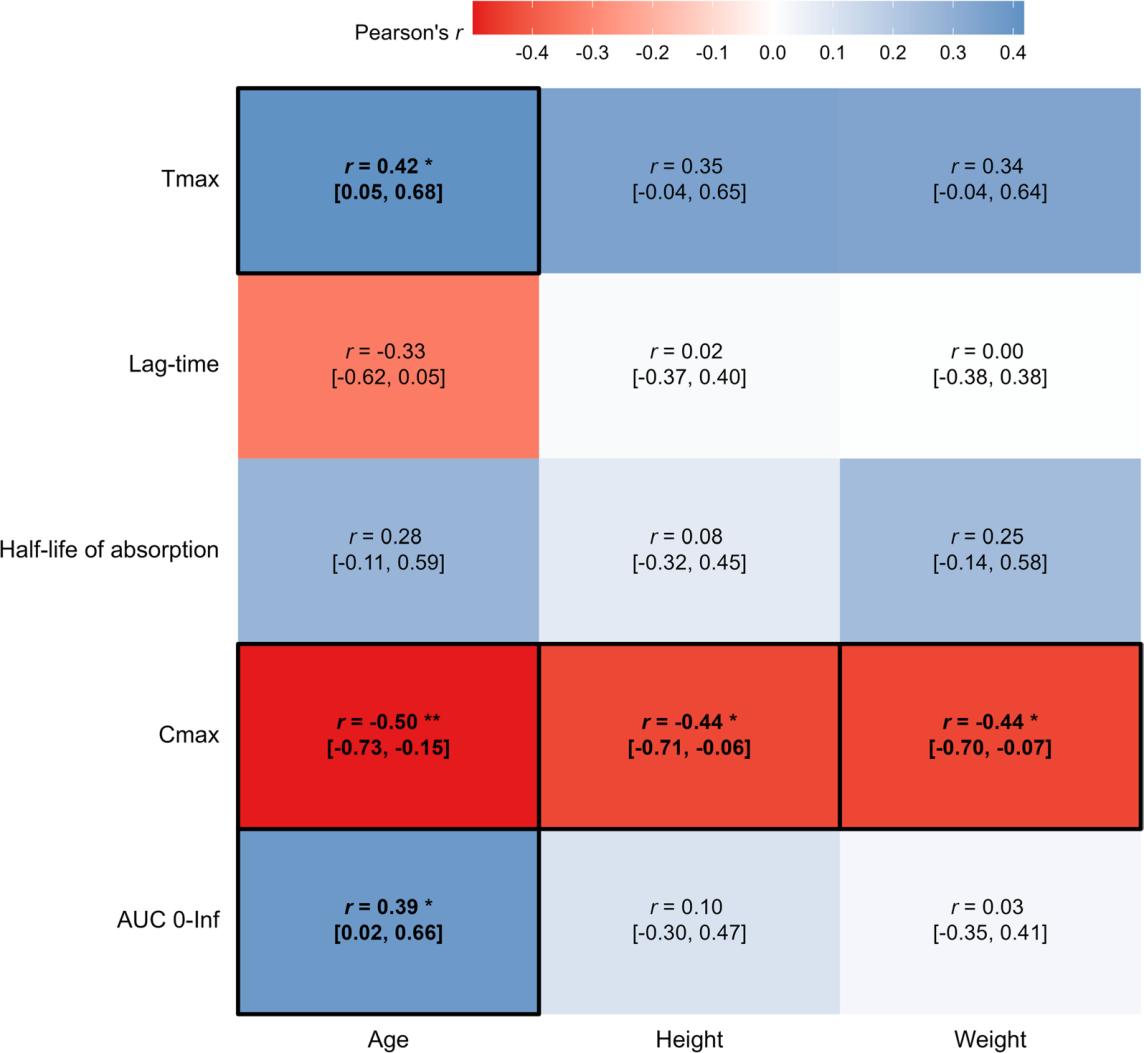
Associations between demographic and pharmacokinetic variables in the reference cohort. Associations between time to maximum concentration (T_max_), lag-time of absorption (T_lag_), half-life of absorption (T_1/2, abs_), dose-normalized maximum concentration (C_max_), dose-normalized area under the curve (AUC _0-Inf_) and age, height, and weight in the reference cohort. Associations are presented as Pearson’s rho (*r*) [95% confidence interval]. * P < 0.05, ** P < 0.01. To aid the reader, values in bold are statistically significant to P < 0.05

When compared with the reference cohort, patients with pineal cysts demonstrated a 0.46 [95% CI: 0.03, 0.88] hours longer absorption lag time (Table 2). In this group, several demographic factors were associated with the pharmacokinetic parameters (Fig. 7); T_max_ and dose-normalized AUC_0-Inf_ were positively associated with age, while dose-normalized C_max_ was negatively associated with age, height and weight.

Neither patients with arachnoid cysts nor patients with spontaneous intracranial hypotension demonstrated any difference in pharmacokinetic parameters of intrathecally administered gadobutrol, compared with the reference cohort (Table 2).

In contrast, patients with idiopathic intracranial hypertension showed a 2.25 [95% CI: 0.74, 3.77] hours shorter absorption half-life when compared with the reference group, indicating a greater CSF to blood clearance of gadobutrol. Additionally, a 3.09 [95% CI: − 5.74, − 0.43] μM h greater dose-normalized AUC_0-∞_ was found compared with the reference cohort (Table 2).

In iNPH patients, compared with the reference cohort, time to maximum concentration was 2.36 [95% CI: 0.30, 4.41] hours longer, and showed a 5.91 [95% CI: 8.18, 3.63] μM h greater mean AUC_0-∞._ Additionally, the lag-time was 0.42 [95% CI: 0.09, 0.74] hours longer compared with reference (Table 2); hence, in iNPH the CSF tracer stays longer within the CSF compartment prior to clearance to blood.

Patients with communicating hydrocephalus demonstrated a 0.19 [95% CI: 0.02, 0.37] μM lower dose-normalized maximum concentration of gadobutrol when compared with the reference group, which was the lowest concentration measured in the included disease categories (Table 2). Even though no statistically significant differences between patients with non-communicating hydrocephalus and the reference cohort were found, most likely due to a low number of subjects in the aforementioned group, time to maximum concentration was numerically higher (12.33 ± 7.17 h), compared to the reference cohort (7.49 ± 4.09 h), as shown in Table 2.

## Discussion

In this work, we present a population pharmacokinetic model applied to intrathecally administered gadobutrol that precisely estimates the clearance from CSF to blood in patients with various diseases. The included patients showed a high degree of inter-individual variability in pharmacokinetic parameters both within and between different disease categories of CSF disturbances.

Up to now, the literature on CSF to blood clearance has been scarce. The presently described model is derived from 1,140 blood samples in 161 patients, referring to plasma levels of gadobutrol measured subsequently to intrathecal injections of predefined quantities. Utilizing positron emission tomography (PET), others [35] previously examined clearance of intrathecal ^99m^Tc-DPTA (technetium-99-diethylene-triamine-pentaacetate) to urine. It also has been demonstrated reduced clearance of a PET ligand from cerebral ventricles to the nasal turbinate in Alzheimer patients [36]. Furthermore, another recent PET study [37] showed reduced clearance of two PET tracers (^18^F-THK5351 and ^11^C-PiB) from ventricular CSF in patients with Alzheimer’s disease, providing support to the hypothesis that impaired clearance of amyloid-β from CSF underlies the amyloid cerebral deposition characterizing Alzheimer’s disease. However, with regard to PET, a drawback is that radioactive ligands provide a radiation dose to the individual [38], have short half-life (about 6 h for ^99m^Tc-DPTA), and the diagnostic process is both expensive and time-consuming.

The most significant observation of the present study is the large inter-individual variation in CSF to blood clearance, as well as the differences between CSF disease. Compared to the reference cohort, patients diagnosed with pineal or arachnoid cysts, and to some degree patients with spontaneous intracranial hypotension, did not present any differences in pharmacokinetics of intrathecally administered gadobutrol. On the other hand, a statistically significant longer lag-time was found in patients with pineal cysts, but no difference in CSF to blood clearance was found. We conclude that on the group level, these categories may possibly reflect the normal variation.

Patients with idiopathic intracranial hypertension, on the other hand, demonstrated a significantly reduced absorption-half life, possibly indicating faster egress of molecules from CSF to blood due to increased ICP. Furthermore, in patients with iNPH, the time to maximum concentration was significantly longer compared to the reference group, and lag-time of absorption was significantly increased. Therefore, in patients with iNPH, the CSF tracer stays longer in the CSF compartment and it requires longer time to reach maximum concentration. The senior authors previously found evidence of reduced CSF turnover in iNPH [23, 39]. In iNPH patients, high grade ventricular reflux of tracer [40] may as well contribute to the increased lag time in these individuals. The CSF to blood clearance of gadobutrol per se was not affected at group level since the absorption half-life or maximum concentration was not different.

We may not from the present data decipher which transport routes gadobutrol follow from the CSF to blood. Emerging evidence points at the role of meningeal lymphatic vessels for molecular egress from CSF to blood, which is supported by findings of reduced clearance of neurotoxic metabolites from CSF when meningeal lymphatic clearance routes are impaired [5]. In humans, the parasagittal dura may be a direct passage route to the meningeal lymphatic structures [24], though molecular efflux from CSF via the cribriform plate seems to be minor [41]. Other possible efflux routes are the cranial and spinal nerve roots [42], and spinal lymphatic pathways [43]. The arachnoid membrane itself has traditionally been considered impermeable to larger molecules [44]. Hence, a CSF tracer study of mice found no signs of tracer propagation beyond the arachnoid layer [45]. Traditionally, it has been thought that CSF egresses via arachnoid granulations to veins, but this view is up to debate [46]. A microscopy study showed endothelial lined gaps and fissures in parasagittal dura of pigs, which might serve as a CSF drainage pathway [47]. In humans, a subset of arachnoid granulations might drain CSF via lymphatic vessels to the venous circulation [48]. Our group showed that the presently used intrathecal tracer gadobutrol enriched in parasagittal dura [24], bone marrow at the skull vertex adjacent or remote to intradiploic dural extensions [25], and in extracranial lymph nodes [49], and demonstrated the feasibility of measuring CSF to blood clearance [27]. The time course of CSF clearance with peak in plasma after 8.60 ± 4.58 h may indicate a major role of the spinal canal given that tracer clearance from CSF peaks to blood occurred far earlier than peak enhancement at the skull vertex [27]. Differences in lag time (T_lag_) might be related to passage capacity within the thecal sac, but we have previously not found differences between groups for time between lumbar injection and first appearance at the cranio-cervical junction, i.e. spinal transit time [50]. We suggest that the meningeal lymphatic vessels are the main route for egress of molecules from CSF, and that meningeal lymphatic impairment may hamper CSF clearance. In this regard, it is of particular interest that evidence from animal and human studies suggest the meningeal lymphatic function deteriorates with increasing age [45, 51], and that impaired meningeal lymphatic function aggravates pathology seen in animal models of Parkinson’s [52] and Alzheimer’s [53] diseases. Experimentally, it was shown that impaired meningeal lymphatic function reduced paravascular influx of macromolecules into the brain, and reduced efflux from the interstitial space [54]. In comparison, we previously found in humans that peak CSF tracer enhancement in human brain and cervical lymph nodes concurred in time, supporting a role of meningeal lymphatic vessels in molecular drainage from CSF [26].

While plasma levels of gadobutrol primarily reflect clearance from CSF along extra-vascular pathways, a minor leakage of tracer through the BBB may to a limited extent contribute to the clearance as ageing as well as neurodegenerative disease may be accompanied with impaired BBB integrity [55, 56]. Evidence of BBB disruption has also been reported for CSF disease such as IIH [57] and iNPH [58]. After entering to the blood, the plasma half-life of gadobutrol in blood is 1.5 h [59].

The present observations may have several clinical implications; we would like to highlight three areas. First, the present observations suggest that assessing CSF to blood clearance adds to characterization of CSF diseases on the individual level. One example is the identification of CSF leakage in individuals with spontaneous intracranial hypotension; it is well established that it may be very difficult to identify the site of CSF leakage [60]. Currently, the visualization of CSF leakage utilizes MRI [60, 61], contrast enhanced computer tomography (CT) myelography [60] as well as intrathecal ^99m^Tc-DPTA nuclear imaging [62], though the risk of not identifying any leakage site is high. A strategy to measure CSF to blood clearance might be expected to aid in identifying individuals with the most pronounced CSF leakage, even though signs of hyper-accelerated clearance could not be shown at group level for the CSF leakage sub-cohort in this study.

Second, direct measurements of CSF to blood clearance might prove useful in preclinical stages of neurodegenerative and dementia disease. Measurements of circulating substances such as amyloid-β and tau may be used for screening purposes, providing an indicative risk of disease [14]. However, direct measures of CSF to blood clearance may be useful in a subset of individuals at risk. In this regard, it should be noted that about ¼ of amyloid-β is cleared via CSF in rodents [63, 64], and a significant amount of tau is excreted via CSF as the majority does not pass across the BBB. For example, mice without dural lymphatic drainage showed significantly reduced excretion of tau [28], and demonstrated a significant association between blood and CSF levels of tau [28]. We here found that the dementia subtype iNPH was characterized with altered pharmacokinetic variables, including longer time to maximum concentration (T_max_), longer lag time (T_lag_) and higher AUC, as compared with reference subjects. However, the difference in AUC may be attributed to the difference in age and renal function compared to the reference cohort.

Third, estimation of CSF to blood clearance may be useful preceding intrathecal drug administration for treatment of neurological disease. Even though it was traditionally thought that a substance within the CSF only passed a few millimeters into the cortical substance [65], intrathecally injected gadobutrol showed brain-wide distribution in humans [22]. Therefore, intrathecal drugs may directly access the entire extra-vascular part of the CNS in contrast to systemically administered substances that are restricted by the BBB [21]. This, however, may depend on the physiochemical properties of drugs. Examples of intrathecal drugs are antisense oligonucleotides [20, 66], such as Spinraza used for spinal muscular atrophy [17, 67], intrathecal chemotherapy, e.g. methotrexate, used for cancer [68, 69], and adeno-associated viral vector-mediated gene-delivery to CNS in amyotrophic lateral sclerosis, dementia disease and spinocerebellar ataxia [16, 70–73]. However, given the high degree of variation in CSF to blood clearance, there is risk of both over- and under-dosage.

Some limitations should be noted. Gadobutrol is administered off-label as it is not approved for intrathecal use. However, here we used gadobutrol in intrathecal doses of 0.10, 0.25 and 0.50 mmol, which have all been proven safe [50, 74]. Toxic effects have previously not been reported for intrathecal gadobutrol in doses of 1.0 mmol or below [75]. We established dose linearity for the range of 0.10–0.50 mmol, and found no difference in the predictive performance of the population pharmacokinetic model between dose levels. As such, for estimating CSF to blood clearance with population pharmacokinetic modelling, an intrathecal dose of 0.10 mmol appears sufficient. Intrathecal gadobutrol is detected in blood with high sensitivity and accuracy; the present detection threshold was about 1.35 nM, well below the observed concentrations, rendering for use of even lower doses. As gadobutrol shares many of the same molecular properties with radiopaque contrast agents, where many are approved for intrathecal use, utility of on-label contrast agents intrathecally for CSF clearance assessment could be explored in future studies.

In this work, the less tangible absorption half-life was used as a surrogate marker for CSF to blood clearance, instead of actual clearance, due to the lack of accurate determinations of individual CSF volume. However, this does not affect the interpretation or accuracy of the results. With regard to the possible normal CSF to blood clearance in healthy people, it may as well be considered a limitation that we included a range of patients spanning multiple defined CSF disturbances. It was, however, beyond the scope of this work to discuss in detail the underlying diagnoses and the clinical significance of each disease category. Additional work on the subject would benefit from the inclusion of individuals without evident neurological disorders, in order to establish a reference value and level of variability in a healthy population. Furthermore, it remains to be determined whether gadobutrol is a valid marker for clearance of other intrathecally administered drugs and endogenous metabolites of interest in disease such as amyloid-β, tau, and α-synuclein.

## Conclusions

In conclusion, this work provides a population pharmacokinetic model of CSF to blood clearance based on 1,140 blood samples from 161 subjects. Our data demonstrates a large degree of inter-individual variability in CSF to blood clearance as well as different clearance profiles across disease categories. CSF clearance function might both be a secondary feature of various neurological diseases, and a primary driver behind disease. As such, extensive clearance may characterize CSF leakage and spontaneous intracranial hypotension, while protracted clearance may be a contributing factor in neurodegenerative diseases. In the therapeutic setting, CSF to blood clearance may prove useful for tailoring dosage of intrathecal drugs, an administration route with prospects of increased utility in the near future.

## Data Availability

The data that support the findings of this study are available from the corresponding author, upon reasonable request.

## Abbreviations

AC: Arachnoid cyst
AUC: Area under the curve
AIC: Akaike’s Information Criteria
BBB: Blood brain barrier
BIC: Bayesian Information Criteria
BMI: Body mass index
C_max_: Maximum concentration
CNS: Central nervous system
CSF: Cerebrospinal fluid
Comm HC: Communicating hydrocephalus
CT: Computer tomography
CV: Coefficient of variations
GFR: Glomerular filtration rate
ICP: Intracranial pressure
IIH: Idiopathic intracranial hypertension
iNPH: Idiopathic normal pressure hydrocephalus
MRI: Magnetic resonance imaging
Non-comm HC: Non-communicating hydrocephalus
PC: Pineal cyst
PET: Positron emission tomography
REF: Reference
RMSE: Root mean squared predictive error
SIH: Spontaneous intracranial hypotension
T_1/2, abs_: Absorption half life
Tlag: Lag-time (of absorption)
T_max_: Time to maximum concentration
